# Anti-inflammatory and antioxidant property of a novel floral mouthwash

**DOI:** 10.6026/97320630019407

**Published:** 2023-04-30

**Authors:** Iram Rafique Pawane, Arvina Rajasekar

**Affiliations:** 1 Department of Periodontics, Saveetha Dental College and Hospitals, Saveetha Institute of Medical and Technical Sciences, Saveetha University, Chennai - 600077, India

**Keywords:** Antioxidant, anti-inflammatory, inflammation, mouthwash, oxidative stress

## Abstract

It is of interest to prepare and evaluate the antioxidant and anti-inflammatory activity of a novel *Clitoria ternatea* and *Matricaria chamomilla* infused mouthwash. The floral extract using the two flowers in dried form was prepared in concentrations of 10µL, 20µL, 30µL, 40µL and 50µL. To assess the antioxidant activity, DPPH assay was done with L-ascorbic acid as the control agent. The anti-inflammatory activity of the floral extract was assessed by the Albumin Denaturation Assay for which diclofenac sodium was used. The absorbance value of the floral extract was determined for each of the concentrations for the antioxidant and anti-inflammatory properties. After 50 µL the prepared extract began to show close absorbance values compared to the standard solutions; at this concentration, the mouthwash was prepared. The preparation and evaluation of the floral extract of *Clitoria ternatea* and *Matricaria chamomilla* infused mouthwash shows promising anti-inflammatory and antioxidant properties and can be used as an adjunct in oral hygiene practices to reduce gingival inflammation. Further clinical studies using the prepared mouthwash are needed to test its efficacy.

## Background:

The maintenance of oral hygiene has been of utmost importance in preventing oral diseases such as dental caries, gingivitis, and periodontitis. Proper tooth brushing techniques, tongue cleaning, flossing, mouth rinses, regular dental checkups and food habits play a central role in the preservation of the oral structures. The most complicated to manage; periodontal disease is the consequence of a sequelae of multiple bacterial and host factors. It was previously hypothesized to be a pathogen-induced disease, but is now known to be a multifactorial one which includes a plethora of uncontrolled immune responses and oxidative stress from reactive oxygen species.[1, 2] This shift in concept opens up a new approach in the management of periodontal disease.

Adjuncts such as anti-inflammatory drugs, antioxidant drugs etc. are effective but in the long term, have adverse effects. Chlorhexidine, which is used as an adjunct to treating periodontal disease, is bacteriostatic (0.2%) and bactericidal (2%) at different concentrations; bi-cationic, positive chlorhexidine molecule interacts with negatively charged phosphate ions on cell walls of pathogens, and has property of substantivity.[3] However, it does have undesired effects such as staining of teeth and prosthesis and taste changes. To counter these, the focus has now been on finding natural agents that are potent anti-inflammatory and antioxidant agents that help combat periodontal disease without any systemic problems.[4]

*Clitoria ternatea* known locally as butterfly pea is a persistent flowering plant from the family Fabaceae, attracting interest in various fields with applications in medicine, agriculture, and the food industry. It has wide applications in traditional medicine for pain relief, inflammation, and fever and as an antidiabetic agent as well as it helps with enhancing cognitive function. C.ternatea has pharmacological properties, phytochemical composition, and active constituents such as anthocyanins which give the flower its vivid blue color. [5, 6, 7, 8, 10] Animal studies have reported that the extracts have not only anti-inflammatory and antioxidant properties but also antiarthritic, antidiabetic, nootropic, analgesic, diuretic, antiasthmatic, antipyretic and wound healing properties.[11, 12]

*Matricaria chamomilla* (M.chamomilla) or commonly known as Chamomile, is a member of the compositae family and is the most commonly used medicinal plant as it has anti-inflammatory, sedative, antimicrobial and antispasmodic properties that are due to the several biologically active compounds such as essential oils (which consist of terpenoids, α-bisabolol, azulenes) and acetylene and polyphenols.[13] Certain constituents present in chamomile such as apigenin glycosides have also demonstrated anticancer properties that have yet to be explored. Our team has extensive knowledge and research experience that has translated into high quality publications [14 15, 16, 17, 18, 19, 20 21, 22, 23]. These promising properties of both floral extracts could aid in the prevention of oral diseases. Hence, the main objective of this study was to prepare and analyze the anti-inflammatory and antioxidant properties of *Clitoria ternatea* and *Matricaria chamomilla* infused mouthwash.

## Material and Methods:

## Preparation of floral extract and mouthwash:

Dried *Clitoria ternatea* and *Matricaria chamomilla* flowers were crushed using a mortar and pestle and weighed. Equal parts of the two prepared floral powders (50 grams each) were mixed and a 5% w/v suspension was prepared in a beaker by adding boiling water. The beaker was placed on a VDRL shaker to constantly shake the solution at 200 rpm for 5 hours and the temperature was maintained at 37° C. After the suspension was cooled, it was filtered using a series of whatman filters and finally through a 0.22-micron filter paper (Millipore, Billerica, MA).

## Antioxidant activity (using the DPPH Radical Scavenging Assay):

The method for determining the antioxidant activity of the novel floral extract consisting of *Clitoria ternatea* and *Matricaria chamomilla* flowers was one reported by Bors et al.[24] Concentrations of the floral extract (10 µl, 20 µl, 30 µl, 40 µl, 50 µl) were prepared and combined with 100 mL of a 100M methanol solution of DPPH (2,2-diphenyl-1- picrylhydrazyl), incubation of the ELISA plate was done at 25°C for 35 minutes. The absorbance of the different solutions was determined at 517 nm. The standard control used for this assay was L- ascorbic acid. The absorbance of the samples and control DPPH scavenging activity (%) was determined by the following formula:

DPPH scavenging activity (%) = [(Control - Sample)/control] x 100.

After triplicate assay was performed, the concentration required for half reduction (IC50) in DPPH activity was plotted. The DPPH assay works on the principle that the antioxidant reacts with 1,1, -Diphenyl-2-picrylhydrazyl, a free radical, in a vivid purple color converts into 1,1-Diphenyl-2-picrylhydrazine, a yellowish color. The degree of the discoloration determines the antioxidant activity of the solution.[25]

## Anti-inflammatory activity (using the Albumin Denaturation Assay):

The method used for determining the anti-inflammatory activity of floral extract consisting of *Clitoria ternatea* and *Matricaria chamomilla* flowers was done by albumin denaturation assay. 0.4 mL of serum albumin (bovine) was mixed with 0.05 mL of the floral extract (of each of the concentrations) and the standard was diclofenac sodium. The pH of the mixture was adjusted to 6.4 by adding 1 M HCl. The suspensions were set at a temperature of 37°C for 15 minutes after which they were heated at 70°C for about 5 minutes. After all the samples were cooled, the absorbance for each of the concentrations was estimated at 660 nm spectrophotometrically. The following formula was used to determine the protein denaturation of each solution:

Protein denaturation (%) = [absorbance value of (control-sample)/absorbance value of control] x 100

The principle is based on the finding that protein denaturation is a causative agent for inflammation. NSAIDs like diclofenac sodium prevent protein denaturation along with cyclooxygenase (COX) enzyme inhibition.[26]

## Results

**Figure 1:**
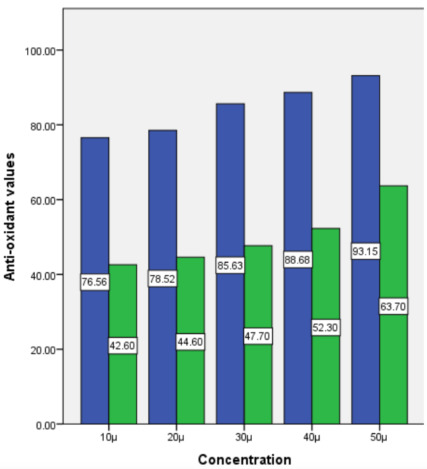
The antioxidative effect of control (blue bar) and floral extract (green bar) at various concentrations (10-50 microliters). At 50 microliters the antioxidant values were the highest for both control and the floral solutions.

**Figure 2:**
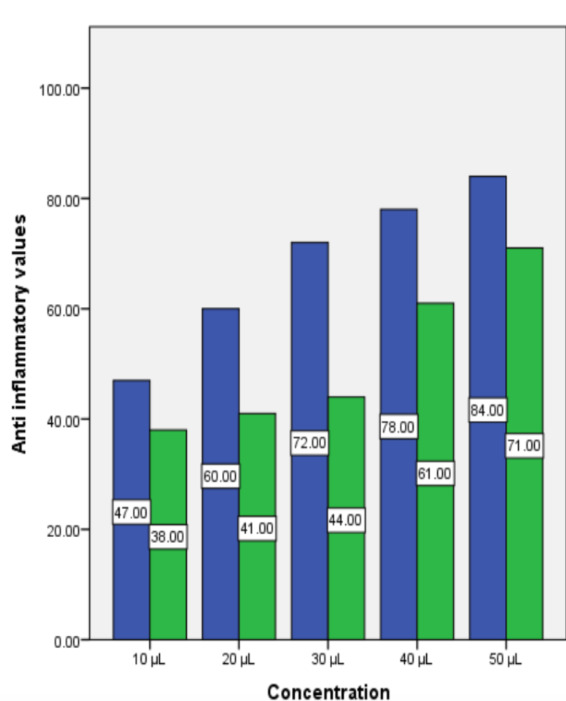
Anti-inflammatory activity representing the percentage of protein denaturation of control and the floral extract at different concentrations. At 50 microliters the anti-inflammatory values were the highest for both control and the floral solutions.

To estimate the antioxidant levels of the floral extract, the absorbance value of the floral extract was determined for each of the concentrations (10-50 µl). After 50 µl the prepared extract began to incrementally show close absorbance values compared to the standard solution, L-ascorbic acid (Figure 1). Determination of the anti-inflammatory activity of the floral extract was recorded similarly. 10 µl- 50 µl incremental concentrations used were compared to diclofenac sodium and spectrophotometrically analyzed at 660nm, after reaching the concentration of 40- 50 µl the extract showed comparable values to diclofenac sodium standard. (Figure 2) From the above finding, 50µL was the most effective concentration for the preparation of the floral mouthwash. To the extract, 2 drops of peppermint extract, 0.3g sucrose, 0.01g sodium lauryl sulphate and 0.5g sodium benzoate was mixed with distilled water.

## Discussion:

From the above study, the synergism of the two potent floral extracts known to have significant medicinal value have shown excellent antioxidant and anti-inflammatory properties. The antioxidant property of the floral extract showed almost comparable antioxidant activity by inhibiting DPPH to the control, L-ascorbic acid, which is a potent antioxidant that neutralizes free radicals. The properties of antioxidation of the *Clitoria ternatea* extract have shown to be protective of canine erythrocytes from oxidative damage induced by AAPH (2,2'-azobis-2-methyl-propanimidamide dihydrochloride) a compound that generates stable free radicals. [27] The erythrocytes were treated with 400µg/mL of *Clitoria ternatea* extract and showed to have high levels of glutathione, overall low levels of protein oxidation and most importantly lipid peroxidation induced by AAPH. One study demonstrated that extracts of *Clitoria ternatea* when used as a stabilizer facilitated the synthesis of magnesium oxide nanoparticles; they found that these nanoparticles exhibited antioxidant properties. [28]

*Matricaria chamomilla* extracts have shown excellent antioxidant properties as well, that have been used in high cholesterol diets in male Wistar rats, and the extract has significantly prevented high levels of superoxide dismutase and plasma malondialdehyde caused due to such diets. [29] A similar study done on male diabetic rats, used 10-20% of *Matricaria chamomilla* extract and demonstrated decreased lipid peroxidation. They also reported an increase in glutathione levels, catalase as well as acetylcholinesterase levels in serum. These studies are in accordance with the study done above, where the combined antioxidant effect of the floral extract has been seen to be comparable to the standard.

*Clitoria ternatea* extracts have excellent anti-inflammatory properties, and as demonstrated in the above done study, its anti-inflammatory effect was documented to be almost comparable to diclofenac sodium at 50µL making it a potent anti-inflammatory. Studies have shown that 400 mg/kg body weight administered orally of *Clitoria ternatea* extracts inhibited induced edema and vascular permeability in rats. The effects were comparable to diclofenac 20 mg/kg oral dosage of diclofenac sodium, an non-steroidal anti-inflammatory drug (NSAID).[30] The anti-inflammatory effects of *Matricaria chamomilla* extract has been reported in a study done on mice macrophages, there was an association of the use of the extract in increased cell viability of macrophages, reducing free radical production e.g. Nitric oxide. It was also noted that there was an induction of IL-10 cytokine production depending on the nature of the extract solution. [29,30, 31] Another study investigated that the extract of *Matricaria chamomilla* extracts inhibit induced edema in rats' feet and mice ears (induced by carrageenan and xylene respectively). Significant inhibition of PGE2 and nitric oxide levels; increase in celiac capillary permeability in mice. [32] In humans it was seen to have caused a stabilized human red blood cell membrane and inhibition of protein denaturation. [33]

## Conclusion:

The preparation and evaluation of the floral extract of *Clitoria ternatea* and *Matricaria chamomilla* infused mouthwash shows promising anti-inflammatory and antioxidant properties and can be used as an adjunct in oral hygiene practices to reduce gingival inflammation. Further clinical studies using the prepared mouthwash are needed to test its efficacy.

## Data Statement:

As this is an in vitro study, no data statement has been added.

## Funding:

Self-funded, this research didn't receive grants from any funding agency in the public, commercial or not-for-profit sectors
